# Comparative Study on Flow-Accelerated Corrosion and Erosion–Corrosion at a 90° Carbon Steel Bend

**DOI:** 10.3390/ma13071780

**Published:** 2020-04-10

**Authors:** Li Zeng, Geng Chen, Hanxin Chen

**Affiliations:** 1 School of Mechanical and Electrical Engineering, Wuhan Institute of Technology, Wuhan 430205, China; li_zeng@wit.edu.cn; 2 Guodian Hanchuan Power Generation Co., Ltd., Hanchuan 431614, China; chengeng0601@126.com

**Keywords:** flow-accelerated corrosion, erosion-corrosion, hydrodynamics, SEM

## Abstract

Electrochemical measurements and surface analysis are performed to comparatively study flow-accelerated corrosion (FAC) and erosion–corrosion (E-C) behavior at a 90° carbon steel bend. The corrosion rates are higher under FAC conditions than those under E-C conditions. For FAC, the corrosion is more serious at the inside wall. However, corrosion is exacerbated at the outside wall under E-C conditions. No erosion scratches are observed under FAC conditions and at the inside wall under E-C conditions, while remarkable erosion scratches appear at the outside wall under E-C conditions. The dominant hydrodynamics affecting FAC and E-C are remarkably different.

## 1. Introduction

Corrosion under fluid flow, which will cause wall thinning and an even perforation of pipelines, is a major issue in oil and gas production [1,2,3]. Corrosion under fluid flow usually gives rise to the premature failure of flow parts and the bankruptcy of oil and gas enterprise, resulting in tremendous pecuniary loss, environmental pollution, and security, lurking peril [4,5]. Particularly, when solid particles are entrained to corrosive flow medium, the pipelines bear erosion and corrosion simultaneously, and erosion–corrosion (E-C) will be aggravated [6,7]. Owing to the synergism between erosion and corrosion, which is the coupling between electrochemical and mechanical processes, it is challenging to authentically grasp the essential origins of the material failure and dependably forecast the lifespan of pipelines [8,9,10]. 

Ordinarily, corrosion under single-phase flow is known as flow-accelerated corrosion (FAC). Mechanical erosion and the interaction between erosion and corrosion under single-phase flow are so small that they can be neglected, while the total weight loss of materials caused by E-C is generally much higher than the sum of pure electrochemical corrosion and pure mechanical erosion owning to the synergistic effect of erosion and corrosion [11,12,13]. E-C usually occurs under two-phase or multiphase flow. The damages aroused by E-C and FAC and corresponding mechanisms are distinctly different since the predominant hydrodynamic characteristics affecting single-phase FAC and multiphase E-C are distinct. However, the impacts of hydraulics on the FAC and E-C have not been adequately understood. Generally, corrosive species rapidly reach metal surfaces, and protective corrosion products are easily damaged and removed away with an increased velocity [14,15]. A high wall shear stress, which is the primary executioner destroying corrosion product films, will be generated between fluids and pipe walls with a high velocity [16,17]. However, other perspectives believe that corrosion products or protective films on steel surfaces are not vulnerable to shear damage, and materials are effectively protected from corrosion attack when the flow velocity is below a certain threshold of the critical flow velocity. When the flow rate is beyond the critical flow rate, the corrosion will be dramatically expedited and steel materials will suffer fatal corrosion failure [12,18]. The presence of suspended sand particles in corrosive fluid causes a more complicated mechanical force exerted by fluid and sand particles. The sediment of sands at the bottom of the pipeline at a low flow rate leads to a corrosion-dominated pattern, and the violent impacts of both fluid and particles at a high flow rate result in erosive wear and the detachment of corrosion products from the steel substrate [19,20,21,22]. Nevertheless, the respective contribution of these two hydrodynamic factors, i.e., fluid and sand particles, to E-C damage still remains ambiguous. Therefore, a comparative study between FAC and E-C is essential to illustrate the intrinsic mechanism of the two different failure modes of materials. 

The bend, which connects pipes together and changes the fluid flow direction, is one of the indispensable components in the oil and gas field. The tremendous changes of the fluid flow regime and sand particles’ movements at bends [23,24] lead to greater FAC and E-C damage in these positions than those in straight pipes and inhomogeneity of FAC and E-C at different places of the bend. However, the distinctness of FAC and E-C behavior and the cardinal hydrodynamics affecting these two types of corrosion damage at the bend is unclear.

In this study, FAC and E-C behavior at a 90° carbon steel bend was comparatively studied. Weight loss was performed to determine the E-C rate. Electrochemical measurements were performed to ascertain the corrosion rate and elucidate the electrochemical corrosion mechanisms under flow environments. Scanning electron microscopy (SEM) was employed to characterize the surface morphology of electrodes under FAC and E-C conditions. The comparison between FAC and E-C behavior at the bend and the predominant hydrodynamics of FAC and E-C were illustrated.

## 2. Materials and Methods

### 2.1. Materials and Media

API X65 carbon steel coupons with an exposed area of 8 mm × 12 mm were used as the specimens to investigate the FAC and E-C. The chemical composition (wt %) of X65 carbon steel is C 0.04%, Si 0.2%, Mn 1.5%, P 0.011%, S 0.003%, Mo 0.02%, and Fe balance. The exposure surface of each specimen was fabricated into the specific curved surface fitting the inner surface of the bend. An iron wire was welded to the backside of each electrode to achieve electric conduction for in situ electrochemical measurements under flowing conditions. The specimens were ground with 800 grit SiC abrasive paper; then, they were cleaned with deionized (DI) water and ethanol, air-dried, and conserved in the desiccator for the flowing tests.

The chemical composition of electrolyte solution is 90.44 g/L NaCl, 2.20 g/L KCl, 0.43 g/L CaCl_2_, 0.43 g/L Na_2_SO_4_, 6.33 g/L MgCl_2_·6H_2_O, and 0.49 g/L NaHCO_3_, which is the formula of the produced water of an oil field. The media were prepared with analytically pure reagent and DI water. The electrolyte solution was deaerated by purging CO_2_ gas (with a purity of 99.95%) for 12 h to reach a CO_2_ saturation level before commencing the test. Then, 100 g sands with the size of 400–500 μm were added into the reservoir prior to the tests. The sand concentration, which was stabilized at 0.29 wt %, was calculated according to the weight of the sands and the volume of electrolyte solution in the pipe (excluding the reservoir). 

### 2.2. Experimental Apparatus

**Figure 1:**
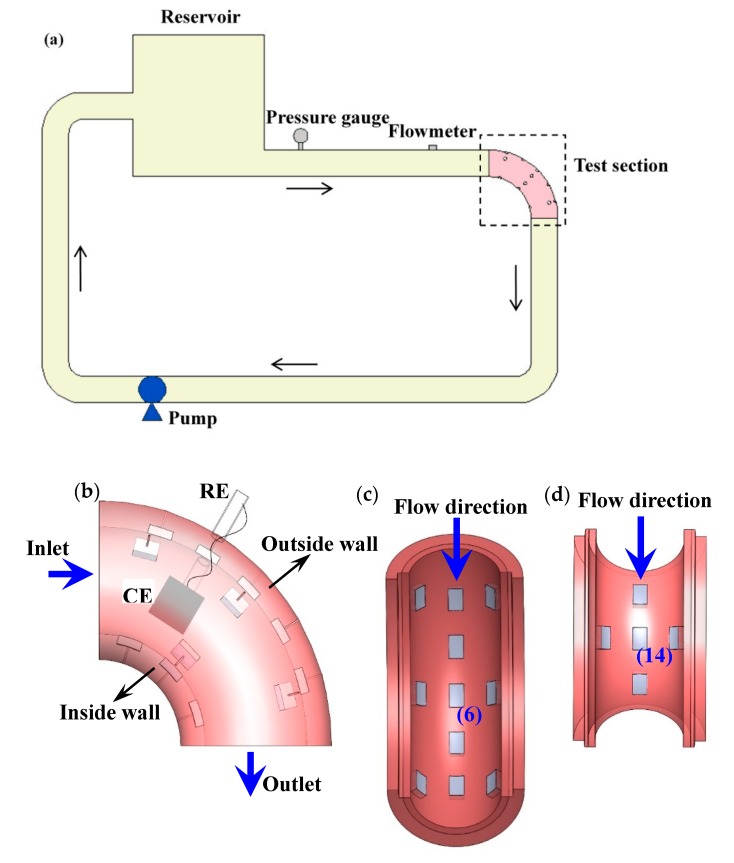
Schematic diagrams of the loop apparatus and bend testing zone for flowing tests: (**a**) loop apparatus; (**b**) bend testing zone; (**c**) array electrodes distribution at the outside wall; (**d**) array electrodes distribution at the inside wall.

A loop apparatus as shown in Figure 1a was employed for flowing tests. A detailed information about the loop apparatus was described elsewhere [25,26]. A total of 11 electrodes at the outside wall and 5 electrodes at the inside wall were encapsulated with silicone glue at the bend testing zone, as shown in Figure 1b–d. The exposed working surfaces were repositioned to be aligned with the inner surface. CO_2_ gas was aerated into the closed loop after the bend testing zone was installed; thus, the electrodes were in a CO_2_ saturation environment before commencing the test. 

### 2.3. Flowing Tests

The flowing tests were conducted for 8 h with the flow rate of 4 m/s, media temperature of 60 °C, and barometric pressure. The total E-C rate of each electrode was achieved by weight loss after the E-C test. The surfaces on which the corrosion products were formed were immersed in the self-made acid solution to remove the corrosion product films. Then, the specimens were rinsed with DI water and ethanol, air-dried, and weighed. The total weight loss of materials during E-C process (corresponding to the E-C rate) is composed of two parts, i.e., corrosion and erosion (corresponding to the corrosion rate of E-C and the erosion rate of E-C, respectively). While under single-phase flow, the loss of materials could be ascribed to corrosion (corresponding to the corrosion rate of FAC) as the mechanical damage is weak during the FAC process. A three-electrode cell composed of array electrodes (WEs), a saturated calomel electrode (RE), and a platinum sheet (CE) was applied to actualize in situ electrochemical measurements at the bend testing zone. Before the electrochemical tests, the cathodic current was not applied on the carbon steel specimen surface for depolarization as the specimens were in a CO_2_ saturation environment. The corrosion rates of E-C and FAC were determined by electrochemical impedance spectroscopy (EIS) at open circuit potential (OCP) with an alternating amplitude of 5 mV at a frequency range from 100,000 Hz to 0.1 Hz during flowing tests. The corrosion rates were ascertained via the Stern–Geary equation and Faraday’s law. Since the weight loss measurements is the average value in the whole test period, the corrosion rate of E-C and FAC was also the average value of EIS at three measurements at different times (at 1 h, 3 h, and 5 h) over all of the testing periods. EIS measurements at the frequency range from 100,000 Hz to 0.01 Hz were yet carried out on typical electrodes 6 (at the outmost wall) and 14 (at the innermost wall) to elucidate the interfacial structures of electrodes. Potentiodynamic polarization measurements were performed on emblematical electrodes 6 and 14 via sweeping potential from −250 mV versus OCP to +250 mV versus OCP with a scan rate of 1 mV/s. For comparison, EIS and potentiodynamic polarization measurements were also conducted on individual electrodes under static states. All of the experiments under flow and static conditions were repeated three times in this work.

### 2.4. Surface Characterization

After the flowing tests, the emblematical electrodes 6 and 14 were rinsed with DI water and ethanol, dried with cooling air, and preserved in the desiccator for surface morphology analysis. Scanning electron microscopy (SEM) was employed to characterize the surface morphologies of the electrodes.

## 3. Results

### 3.1. Total E-C Rate after E-C Test and Corrosion Rate after Flowing Test 

**Figure 2:**
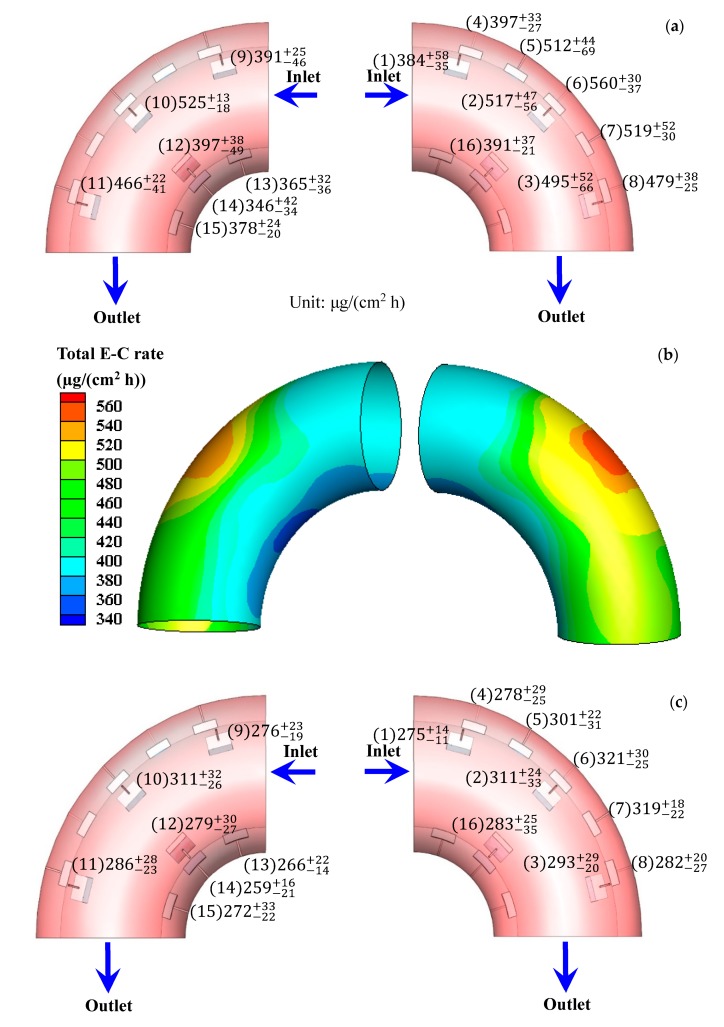

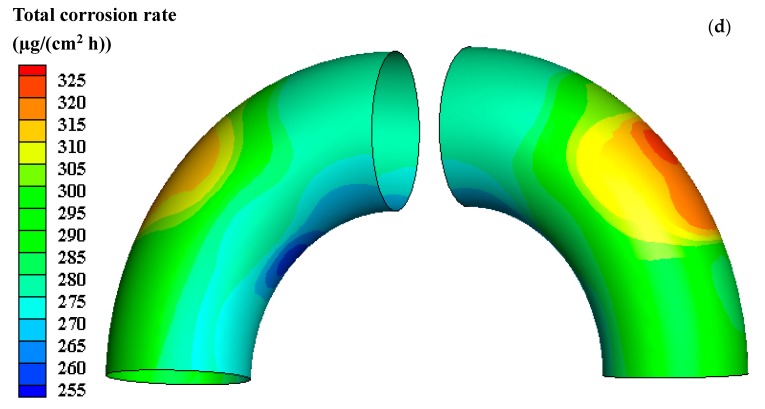
(**a**) Distribution of the total erosion–corrosion (E-C) rate after the E-C test; (**b**) contours of the total E-C rate after the E-C test; (**c**) distribution of the total corrosion rate after the E-C test; (**d**) contours of the total corrosion rate after the E-C test.

**Figure 3:**
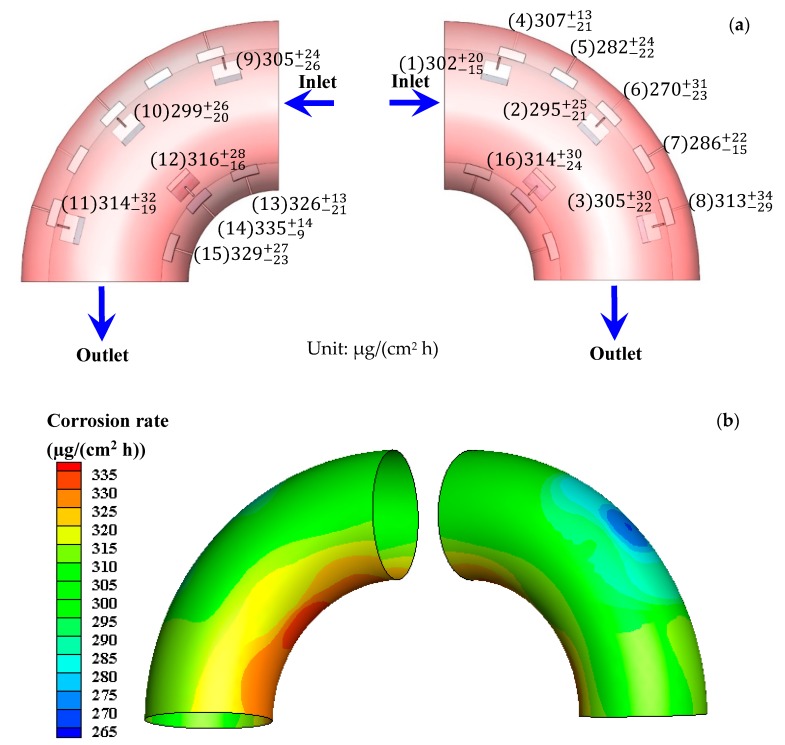
(**a**) Distribution of corrosion rate after the flow-accelerated corrosion (FAC) test and (**b**) contours of corrosion rate after FAC test.

The total E-C rate and total corrosion rate obtained after the E-C test are shown in Figure 2. It can be seen that both the total E-C rate and total corrosion rate are higher at the outside wall of the bend. The values rise first and then fall in the direction of flowing at the outside wall. Nevertheless, the values decrease first and then increase in the direction of flowing at the inside wall. The highest values (559.9 µg/(cm^2^ h) of the total E-C rate and 321.2 µg/(cm^2^ h) of the total corrosion rate) and the lowest values (346.1 µg/(cm^2^ h) of the total E-C rate and 258.7 µg/(cm^2^ h) of the total corrosion rate) appear at the outermost side and the innermost side, respectively. According to our previous work [5], the highest value (182.3 µg/(cm^2^ h)) and the lowest value (45.6 µg/(cm^2^ h)) of the pure erosion rate also appear at the outermost side and the innermost side, respectively. The value of the total E-C rate is much higher than those of the total corrosion rate and pure erosion rate for the electrode in the same position, indicating a synergetic effect between corrosion and erosion under E-C conditions. The distribution of the corrosion rate after the FAC test is shown in Figure 3. The values are higher at the inside wall of the bend, which is contrary to the distributing disciplinarian of the total corrosion rate under E-C conditions. The values drop first and then rise in the orientation of flowing at the outside wall. However, the values increase first and then decrease in the orientation of flowing at the inside wall. The highest value (334.9 µg/(cm^2^ h)) and the lowest value (270.0 µg/(cm^2^ h)) appear separately at the innermost side and the outermost side. The distributions of the corrosion rate at the bend under E-C conditions are distinctly different from the corrosion rate distributions under FAC conditions. Furthermore, the corrosion rates under single-phase flow are not lower than the values under two-phase flow. Besides, the corrosion rates in the flowing state are greatly enhanced in comparison with the corrosion rate of 131.1 µg/(cm^2^ h) under static conditions. The high corrosion rates should be attributed to the violent disturbance of fluid or sand particles, which facilitates the mass transport of corrosive species or removes the corrosion product or film on the electrode surface. 

### 3.2. Electrochemical Impedance Spectroscopy Measurements

**Figure 4:**
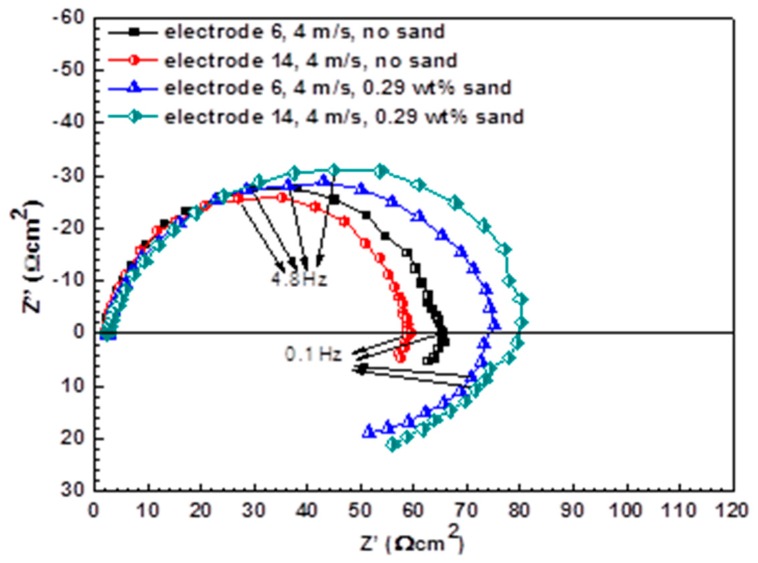
Nyquist plots under FAC and E-C conditions.

**Figure 5:**
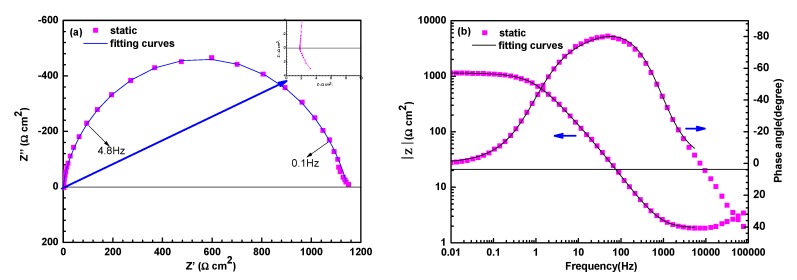
Nyquist (**a**) and Bode (**b**) plots of electrodes under the static state.

Figure 4 shows the Nyquist plots of electrodes under FAC and E-C conditions after 7 h of exposure. The Nyquist plots under flowing conditions are characteristic of the capacitance arc at high frequency and the inductance arc at low frequency. The capacitance arc should be ascribed to double-layer capacitance and charge transfer resistance, and the inductance arc should be imputed to adsorbed intermediate products, which are instable during the dissolution of steel [12,27]. However, the Nyquist plots of electrodes in the static state as shown in Figure 5a are featured in a squashed capacitance arc with one capacitance arc in high frequency and one capacitance arc in low frequency merging together. The arc of high frequency should be attributed to corrosion products formed on the steel surface, and the arc of low frequency should be associated with charge transfer during the electrochemical corrosion process [28]. An inductive semicircle at the frequency range from 100,000 Hz to 9600 Hz should be ascribed to the stray inductance from the electrochemical station [29]. As the iron wire was short (the length was about 10 cm) and was connected to the electrochemical station in a straight (not winding) manner, there should be little possibility to cause the stray inductance from the simple electrolytic cell. The corresponding Bode plots of electrodes in the static state are shown in Figure 5b. It is seen that the Bode plots are characterized by a wide phase angle peak (two time constants) at the negative phase angle range and a phase angle valley corresponding to one time constant at the positive phase angle range. The wide phase angle peak corresponds to the two merged capacitive semicircles, and the phase angle valley at high frequency is related to the inductive semicircle in the Nyquist plots.

**Figure 6:**
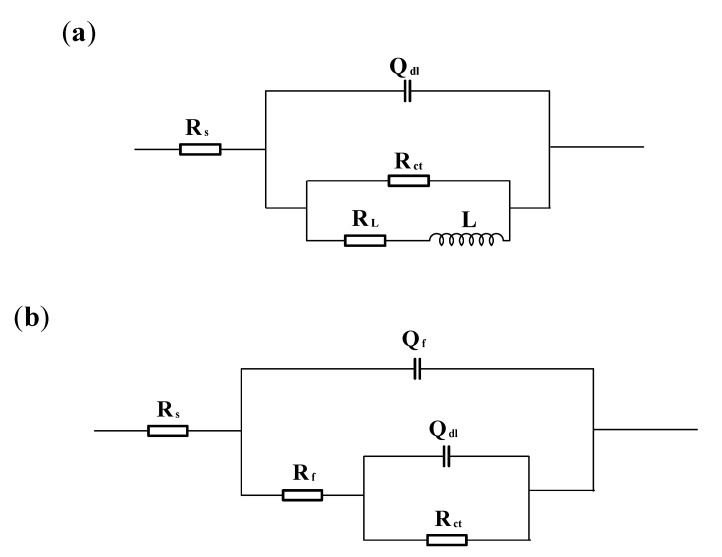
Electrochemical equivalent circuits: (**a**) under flow conditions; (**b**) under static-state conditions.

Electrochemical equivalent circuits as shown in Figure 6 are employed for fitting the EIS data to dissect correlative impedance parameters. Among various parameters, *R*_s_ represents the solution resistance; *Q*_dl_ represents the constant phase element reflecting double-layer capacitance; *R*_ct_ represents the charge transfer resistance; *R*_L_ represents the inductance resistance; *L* represents the inductance; *Q*_f_ represents the constant phase element reflecting the capacitance of the corrosion products’ film; and *R*_f_ is the film resistance. Considering the contribution of geometric characteristics such as a rough or heterogeneous electrode surface to EIS, Q rather than capacitance is proposed to compensate for the non-homogeneity. *Q* reflects the capacitance (*C*) according to Equation (1) [30,31]:

(1) $$C=\frac{{\left(Q\cdot R\right)}^{\frac{1}{n}}}{R}$$

where *R* is the resistance and *n* is the dispersion coefficient. Therefore, *Q*_dl_ and *Q*_f_ are used to reflect the double-layer capacitance and the capacitance of the corrosion products’ film, respectively. The fitted parameters for EIS under flowing and static conditions are listed in Table 1 and Table 2. The charge transfer resistance *R*_ct_ under single-phase flow is lower than that under two-phase flow. For single-phase flow, the *R*_ct_ at the innermost side is less than that at the outermost side. Nevertheless, the *R*_ct_ at the innermost side is higher than that at the outermost side when sands are entrained in the liquid. The value of *R*_ct_ is much larger than that of *R*_f_ under static-state conditions, because the corrosion process is largely controlled by electron transfer between the substrate and the corrosion product film, which is known to be rate-limiting in electrochemical reactions with compact corrosion products under static-state conditions [32]. The *R*_ct_ in the flowing state is much smaller in comparison with that in the static state.

### 3.3. Polarization Curves Measurements

**Figure 7:**
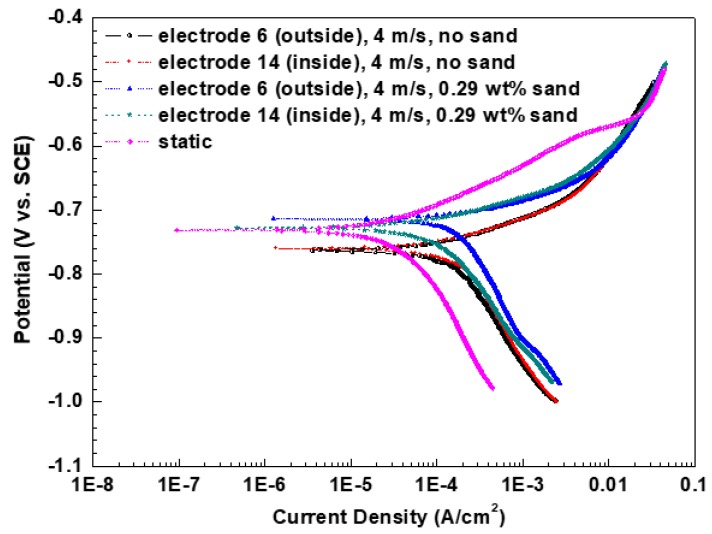
Potentiodynamic polarization curves under flow and static conditions.

Figure 7 shows the potentiodynamic polarization curves under flow and static conditions. For the single flow, the anodic current densities are approximately the same but the cathodic current densities are distinguishing at different positions. The cathodic current density at the inside wall is higher than that at the outside wall, which results in the rise of corrosion current density. Nevertheless, in the presence of sands, both the anodic current density and cathodic current density at the outside wall are bigger than those at the inside wall; as a result, the corrosion current density is higher at the outside wall. The electrochemical parameters obtained by fitting the experimental data are illustrated in Table 3. The corrosion current density at the inside wall is higher under single-phase flow, while the value is bigger at the outside wall under two-phase flow. By comparison, the corrosion current densities under flowing conditions are much bigger than that (3.34 × 10^−5^ A/cm^2^) in static-state conditions. Furthermore, the maximum and minimum values under single-phase flow are larger than those under two-phase flow, respectively.

### 3.4. SEM Surface Morphology

**Figure 8:**
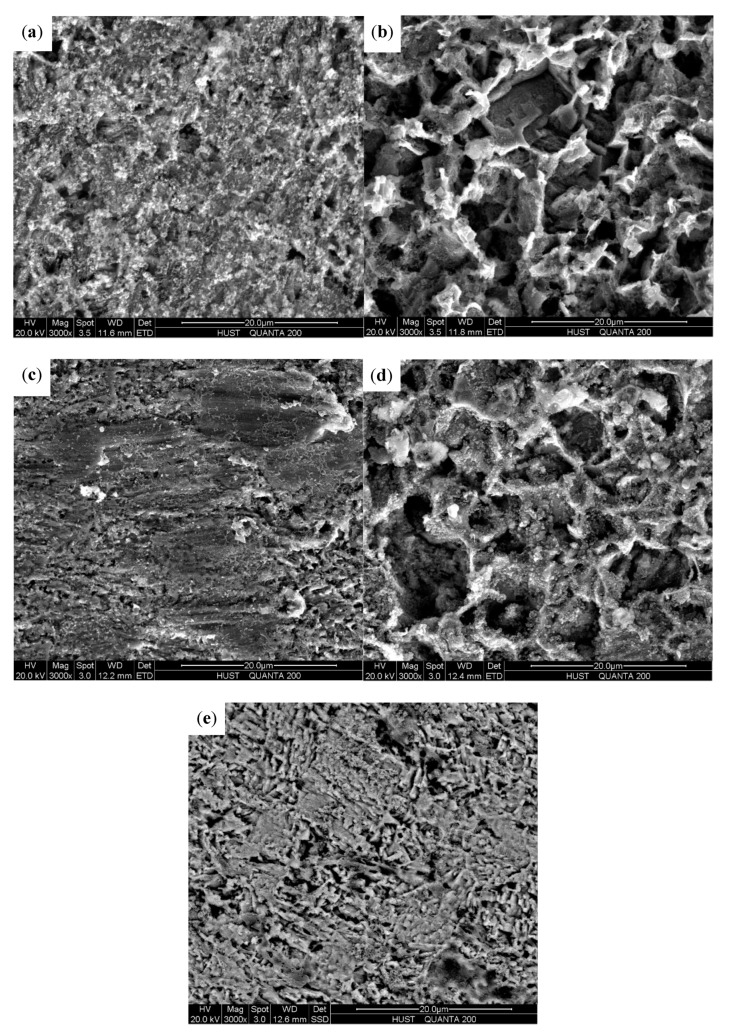
SEM surface morphologies of electrodes under flow and static conditions: (**a**) electrode 6 (outside), FAC test; (**b**) electrode 14 (inside), FAC test; (**c**) electrode 6 (outside), E-C test; (**d**) electrode 14 (inside), E-C test; (**e**) under static-state conditions.

Figure 8 shows the SEM surface morphologies of electrodes under flow and static conditions. Since the characterization of corrosion products is not the main point in this study, the cross-section of the corrosion products was not observed. Only the SEM surface observation was carried out. No erosion scratches but corrosion products are observed under single-phase flow and static-state conditions. The corrosion products under single-phase flow are much looser and more porous in comparison with those in static-state conditions. Particularly, under single-phase flow, the corrosion products at the inside wall are less dense and polyporous compared with those at the outside wall. However, obvious scour scars accompanied with corrosion products are present at the outside wall under two-phase flow. No wear scars but porous corrosion products are observed at the inside wall in the presence of sands. Furthermore, the corrosion product layers at the inside wall under single-phase flow are looser compared with those at the same position under two-phase flow, indicating that much smoother pathways are furnished for corrosion species to permeate through during the FAC process. As a result, it is much easier for the corrosion species to reach the surface of metal substrates, thus facilitating the corrosion reaction process. The presence of the corrosion products on the X65 carbon steel surface, which is of low conductivity and poor crystalline, would influence the quality of the SEM images; even gold was sprayed on the surface of samples. The quality of the used SEM images satisfied the sharpness for comparison of the compactness of corrosion products under different conditions and characterization of the appearances of wear scars at the outside wall of the bend under E-C conditions.

## 4. Discussion

### 4.1. The Effect of Hydrodynamics on the Corrosion Behavior under Single-Phase and Two-Phase Flow

The total E-C rate and total corrosion rate are higher at the outside wall under two-phase flow, while the corrosion rate is higher at the inside wall under single-phase flow (Figure 2 and Figure 3). The corrosion rates during the FAC process are not less than those during the E-C process. Evidently, the FAC and E-C behavior are vastly different. The only distinction is that FAC is in the absence of sands, yet E-C is in the presence of sands. The sands entrained in the liquids substantially alter the hydrodynamics at the bend, leading to quite different flowing corrosion behavior under single-phase and two-phase flow.

It is acknowledged that carbon steel possesses an emblematical microstructure of ferrite (Fe) and carbide (Fe_3_C). The potential of Fe_3_C is more positive in regard to Fe, thus creating a galvanic cell between Fe and Fe_3_C spontaneously. Under single-phase flow, the anodic phase Fe is readily dissolved while the cathodic phase Fe_3_C remains on the steel surface as the impact fracture of a single fluid is inadequate to flush away the Fe_3_C residues. The lax and poriferous residues, which protrude from the surface, are unprotective for the metal substrate, serving as the cathodic phase on one side, and on the other providing fluent channels for corrosive ions transfer. As a consequence, the corrosion of carbon steel is expedited. The size and amount of Fe_3_C with poor crystalline were measured by XRD (X-ray diffraction), but the amount is too low to be detected, and only Fe was detected. Therefore, the significant effect of Fe_3_C on the corrosion behavior of carbon steel was only supported by other researchers [33,34].

Nevertheless, under two-phase flow, the violent sand impact scours off the non-dense corrosion products along with cathodic phase Fe_3_C, even the metal substrates; thus, the corrosion process is inversely retarded. Notably, as E-C is caused by both electrochemical corrosion and mechanical erosion and there is a synergistic effect between corrosion and erosion, the total material loss in the presence of sands is much higher compared with FAC, although the corrosion is worse in the absence of sands. Furthermore, the kinetics of the electrochemical reaction are remarkably different during the FAC and E-C process from the polarization curves measurements (Figure 7). Under single-phase flow, the liquid flowing at the bend predominantly facilitates the mass transfer process of the cathodic reaction; thus, a higher mass transfer rate of cathodic reaction with increased velocity will promote the corrosion of electrodes. However, the situation is distinct when sands are carried into the liquid. The movement of sands at the bend plays the leading role in the corrosion process. On the one hand, the sand impacts damage the loose corrosion products, exposing the fresh metal substrates. Although the immediate mechanical wear is aggrandized, the corrosion of electrodes is dramatically inhibited due to the removal of non-protective corrosion products. On the other hand, the perturbation of sands in fluids can also expedite the mass transfer of the cathodic reaction, thus increasing the cathodic reaction rate. By contrast, the main effect of the sand impacts derives from the destruction of the porous corrosion product layers and the mechanical erosion of metal substrates. As a consequence, severe E-C is generated, but the corrosion is weaker in comparison with that during the FAC process, which is consistent with the lower charge transfer resistances under single-phase flow than those under two-phase flow (Figure 4). From the SEM (Figure 8), the corrosion products are looser and more porous at the identical positions in the absence of sands compared to those with sands, demonstrating that the corrosion product layers are less protective for the metal substrates under single-phase flow. Therefore, the corrosion under single-phase flow is more serious than that under two-phase flow.

### 4.2. The Effect of Hydrodynamics on the FAC and E-C Behavior at the Bend

**Figure 9:**
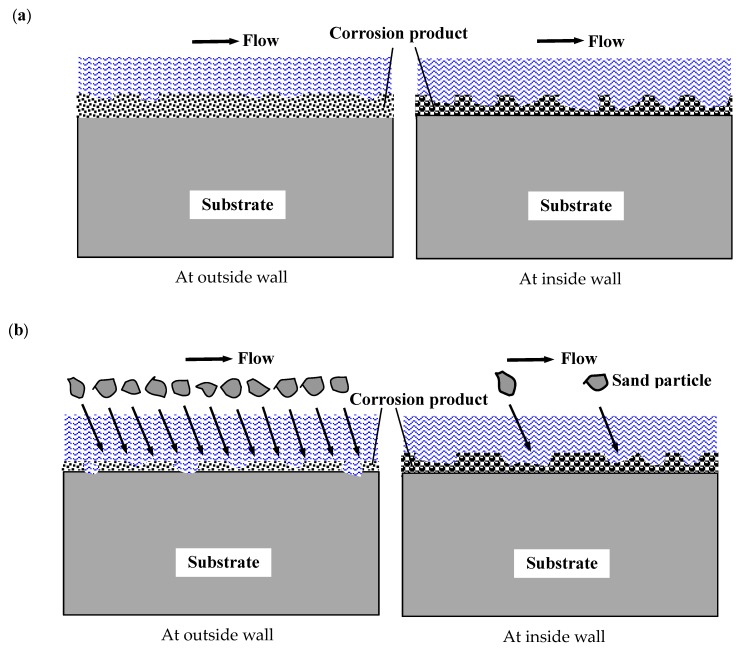
Schematic of the electrodes in different positions of the bend under flow conditions: (**a**) under FAC conditions; (**b**) under E-C conditions.

Our previous investigation shows that the wall shear stress is generally higher at the inside wall than at the outside wall at the velocity of 4 m/s. The maximum value of shear stress (55.0 Pa) appears at the innermost side (Electrode 14), while the minimum value (16.0 Pa) appears at the outermost side (Electrode 6) [17,35]. The accelerated flow rate and wall shear stress at the inside wall of the bend lead to the high turbulence and numerous swirls under single-phase flow. Due to the centrifugal effect at the bend, the pressure is higher at the outside wall, whereas the lower pressure appears at the inside wall. According to the Bernoulli’s principle, the missing pressure potential energy is transformed into the kinetic energy at the inside wall. Furthermore, the squeezing effect of the fluid is intensified at the inside wall because of the centrifugal force, and the flow rate can also be increased. Thus, the wall shear stress as well as the impact energy is aggrandized. Consequently, the corrosion products at the inside wall are partly destroyed, which is ascribed to the preferential dissolution of ferrite in X65 carbon steel under the more severe impact of fluid, leaving the retained Fe_3_C residues on the surface, which is porous under the used magnification, as shown in Figure 8a,b. The typical morphology under flowing conditions is in accordance with the results of other researchers [33,34]. The retained Fe_3_C residues, which have poor protection for the corrosion of the substrate, act as the cathodic phase and promote the corrosion reaction. A schematic of the FAC and E-C of the electrodes in different positions of the bend is shown in Figure 9. In addition, the unprotective corrosion products can also contribute to the rapid mass transfer of the cathodic reaction and thus facilitate the corrosion of carbon steel. At the outside wall, the small flow rate and wall shear stress construct a relative secure circumstance for the corrosion products to remain intact and dense on the surface of the electrodes, thus decreasing the mass transfer of the cathodic reaction and retarding the corrosion process.

However, under two-phase flow, the influence of the sand impacts predominates the hydrodynamics at the outside wall of the bend. The particles move forwards in the upstream straight pipe and impact the outermost side immediately at the turning bend due to the inertia action. Nevertheless, the particles slide along the outer wall, and the abrasion wear occurs at the second-half of the bend. Additionally, the liquid film at these positions is much thicker, which provide a cushioning effect on the particle impact. As a result, the destruction of the material is weakened. In comparison, quite a few sands present at the inside wall. Massive particles impact the outer wall and reflect inward, which is caused by the secondary flow. However, these particles could hardly arrive at the inside wall, as the majority of particles will collide with the liquid and the particles of the main stream moving forward to the downstream straight pipe. Consequently, this part of the particles will be intercepted before impacting the inside wall. The continuous impacts of dense sands at the outside wall [26] will thin the corrosion product layers and even expose the naked fresh substrates to corrosive fluids, greatly promoting the mechanical erosion and electrochemical corrosion in these positions. However, a small quantity of sands play a negligible role in destroying the corrosion products at the inside wall. The situation is similar to that under single-phase flow at the inside wall. The flow disturbance has insufficient power to break the corrosion product, leaving loose and unprotective corrosion product layers on the surface. Therefore, the hydrodynamics at the bend during the FAC and E-C processes work significantly different, resulting in different FAC and E-C behavior in different positions of the bend.

## 5. Conclusions

The corrosion is more severe under single-phase flow than that under solid–liquid two-phase flow. During the FAC process, the fluid flowing leaves the non-protective cathodic phase on the surface and facilitates the mass transfer process, thus promoting the corrosion of carbon steel. However, during the E-C process, the sand impacts mainly destroy the loose corrosion products, and the corrosion is mitigated compared with that under FAC conditions, although the total E-C is drastically enhanced.

The corrosion is aggravated at the inside wall in comparison with that at the outside wall under FAC conditions, while the corrosion and E-C is worse at the outside wall under E-C conditions. The hydrodynamics in the absence and presence of sands take markedly different effects at the bend.

## Figures and Tables

**Table 1:** Electrochemical parameters fitted from the electrochemical impedance spectroscopy (EIS) data of typical array electrodes 6 (outside) and 14 (inside).

Electrode	Sand Concentration (wt %)	*R*_s_ (Ω·cm^2^)	*Q*_dl_ (×10^−4^Ω^−1^cm^−2^ s^n^)	n	*R*_ct_ (Ω cm^2^)	*R*_L_ (Ω cm^2^)	*L* (H/cm^2^)
Electrode 6	0	2.24	7.94	0.92	62.86	126.10	7171
Statistical errors (%)		3.87	5.16	1.82	7.33	17.94	6.03
Electrode 14	0	2.16	8.48	0.93	57.17	175.70	6558
Statistical errors (%)		5.18	3.61	1.42	6.29	18.56	7.02
Electrode 6	0.29	2.37	7.46	0.89	71.48	67.42	846
Statistical errors (%)		7.29	4.81	0.75	6.37	16.42	15.87
Electrode 14	0.29	2.34	6.08	0.85	78.59	81.64	768
Statistical errors (%)		5.86	6.67	1.95	7.92	13.09	18.85

**Table 2:** Fitted parameters for the EIS of the electrode after corrosion under static-state conditions.

	*R*_s_ (Ω cm^2^)	*Q*_f_ (×10^−4^Ω^−1^cm^−2^ s^n^)	*n*_1_	*R*_f_ (Ω cm^2^)	*Q*_dl_ (×10^−4^Ω^−1^cm^−2^ s^n^)	*n*_2_	*R*_ct_ (Ω cm^2^)
Calculated values	1.03	1.06	0.99	222.30	1.68	0.70	924.40
Fitting errors (%)	1.22	6.44	0.84	8.72	10.41	4.40	9.16
Statistical errors (%)	7.78	14.93	0.68	5.91	13.25	3.74	10.26

**Table 3:** Potentiodynamic polarization parameters of typical array electrodes 6 (outside) and 14 (inside) under flow and static conditions.

Electrode	Velocity (m/s)	Sand Concentration (wt %)	E_corr_ (V vs. SCE)	ba(V/dec)	bc(V/dec)	I_corr_ (A/cm^2^)
Electrode 6	4	0	−0.763	0.079	−0.200	1.37 × 10^−4^
Statistical errors (%)			0.74	4.18	1.99	6.85
Electrode 14	4	0	−0.760	0.080	−0.227	1.72 × 10^−4^
	Statistical errors (%)		1.05	4.60	2.51	7.31
Electrode 6	4	0.29	−0.714	0.096	−0.222	1.58 × 10^−4^
	Statistical errors (%)		1.59	5.92	2.10	8.14
Electrode 14	4	0.29	−0.728	0.073	−0.165	8.86 × 10^−5^
	Statistical errors (%)		1.33	5.88	3.44	1.51
	0	0	−0.731	0.063	−0.202	3.34 × 10^−5^
	Statistical errors (%)		0.82	3.07	1.81	2.00
